# EPA-Enriched Phospholipids Alleviate Renal Interstitial Fibrosis in Spontaneously Hypertensive Rats by Regulating TGF-β Signaling Pathways

**DOI:** 10.3390/md20020152

**Published:** 2022-02-19

**Authors:** Hao-Hao Shi, Ling-Yu Zhang, Li-Pin Chen, Jin-Yue Yang, Cheng-Cheng Wang, Chang-Hu Xue, Yu-Ming Wang, Tian-Tian Zhang

**Affiliations:** 1 College of Food Science and Engineering, Ocean University of China, Qingdao 266003, China; shihaohao@stu.ouc.edu.cn (H.-H.S.); zhanglingyu@jmu.edu.cn (L.-Y.Z.); clp@stu.ouc.edu.cn (L.-P.C.); yangjinyue@stu.ouc.edu.cn (J.-Y.Y.); wangchengcheng@stu.ouc.edu.cn (C.-C.W.); xuech@ouc.edu.cn (C.-H.X.); wangyuming@ouc.edu.cn (Y.-M.W.); 2 College of Food and Biological Engineering, Jimei University, Xiamen 361021, China; 3 Laboratory for Marine Drugs and Bioproducts, Pilot National Laboratory for Marine Science and Technology (Qingdao), Qingdao 266237, China

**Keywords:** EPA, phospholipid, blood pressure, nephropathy, interstitial fibrosis

## Abstract

Hypertensive nephropathy is a chronic kidney disease caused by hypertension. Eicosapentaenoic acid (EPA) has been reported to possess an antihypertensive effect, and our previous study suggested that EPA-enriched phospholipid (EPA-PL) had more significant bioactivities compared with traditional EPA. However, the effect of dietary EPA-PL on hypertensive nephropathy has not been studied. The current study was designed to examine the protection of EPA-PL against kidney damage in spontaneously hypertensive rats (SHRs). Treatment with EPA-PL for three weeks significantly reduced blood pressure through regulating the renin–angiotensin system in SHRs. Moreover, dietary EPA-PL distinctly alleviated kidney dysfunction in SHRs, evidenced by reduced plasma creatinine, blood urea nitrogen, and 24 h proteinuria. Histology results revealed that treatment of SHRs with EPA-PL alleviated renal injury and reduced tubulointerstitial fibrosis. Further mechanistic studies indicated that dietary EPA-PL remarkably inhibited the activation of TGF-β and Smad 3, elevated the phosphorylation level of PI3K/AKT, suppressed the activation of NF-κB, reduced the expression of pro-inflammatory cytokines, including IL-1β and IL-6, and repressed the oxidative stress and the mitochondria-mediated apoptotic signaling pathway in the kidney. These results indicate that EPA-PL has potential value in the prevention and alleviation of hypertensive nephropathy.

## 1. Introduction

Hypertension is a common disease that seriously endangers public health [1]. Hypertensive nephropathy is one of the most common chronic complications of hypertension, which is often characterized by renal interstitial fibrosis and the eventual loss of renal function [2,3]. Renal interstitial fibrosis mainly reflects tubular atrophy, interstitial leukocyte infiltration, and increased interstitial matrix deposition [4]. Epidemiological studies revealed that hypertensive nephropathy is closely related to hemodynamics, vascular endothelial dysfunction, inflammation, apoptosis, oxidative stress, to name a few [5]. The current treatment of hypertensive nephropathy mainly uses calcium channel blockers, angiotensin-converting enzyme inhibitors, angiotensin II receptor blockers, and other specific drugs in clinical practices [1]. The long-term drug treatment for patients with hypertension can easily lead to unpleasant side effects. Reports from clinical studies show that that nutritional intervention has become a research hotspot in alleviating hypertensive nephropathy [6,7,8]. Therefore, it is of great clinical significance to identify a natural active compound with fewer adverse effects for ameliorating hypertensive nephropathy.

Eicosapentaenoic acid (EPA), as an omega-3 long-chain polyunsaturated fatty acid (ω-3 PUFA), is widely distributed in the body of multifarious marine products. Notably, EPA exhibits many important physiological activities, which has been considered by investigators to be beneficial in the prevention or treatment of several diseases, such as alleviating cardiovascular disease, improving cognitive function, and lowering blood pressure [9,10,11]. EPA is mainly sold as ω-3 PUFA supplements in a concentrated form as ethyl esters (EE) or triacylglycerol (TG). Interestingly, in recent years, phospholipid (PL) forms containing EPA has been reported to have more significant physiological activity compared with the TG or EE forms [12]. We have previously demonstrated that EPA-enriched phospholipids (EPA-PL) can alleviate metabolic syndrome by regulating the expression of genes associated with the lipid metabolism in ApoE^−/−^ mice [13]. Our previous study also found that EPA-PL exhibited positive protective effects against obesity-induced renal injury [14]. Moreover, EPA-PL had a significant therapeutic effect against vancomycin-induced acute kidney injury [15]. Furthermore, dietary EPA and docosahexaenoic acid (DHA) had a positive antihypertensive effect through the regulation of the renin–angiotensin system in rats with hypertension [7,9,11]. However, the true effects of EPA-PL on hypertension and hypertensive-induced renal interstitial fibrosis have still not been uncovered.

In the present study, a spontaneous hypertensive rat (SHR) model was used to determine the effects of EPA-PL in attenuating elevated systolic blood pressure (SBP), diastolic blood pressure (DBP), and mean artery pressure (MAP) by the renin–angiotensin system. Meanwhile, we evaluated the effect of EPA-PL on renal interstitial fibrosis in rats with hypertensive nephropathy through analyzing the transforming growth factor-β (TGF-β)/Smad 2/3-meditated tubulointerstitial fibrosis, the PI3K/Akt/NF-κB signaling pathways, oxidative stress, and the mitochondria-mediated apoptosis signaling pathway.

## 2. Results and Discussion 

### 2.1. The Change in Blood Pressure in SHRs after Single Oral Administration of EPA-PL

**Figure 1:**
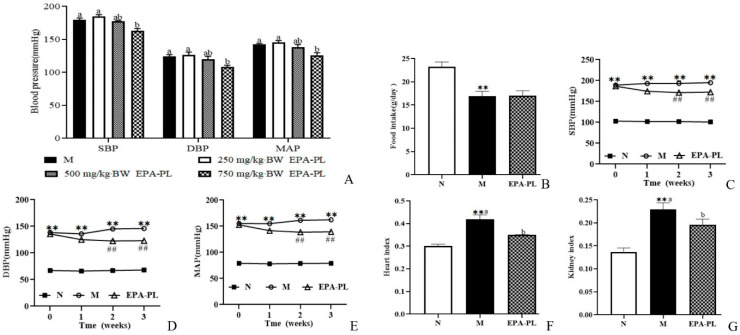
The change in blood pressure in rats after treatment with different doses of EPA-PL by single oral administration (**A**); the effects of dietary EPA-PL on the food intake in rats (**B**); the change in blood pressure in rats with long-term feeding of EPA-PL (**C**–**E**); the effects of dietary EPA-PL on the index of heart and kidney in rats (**F**,**G**). All data were expressed as mean ± S.E.M. for 6 rats. ** Significantly different compared to control group (*p* < 0.01), ^##^ Significantly different compared to the model group in the long-term experiment (*p* < 0.05). Different letters represent significant differences among experimental groups, and means with the same letter were not significantly different (*p* < 0.05). Abbreviations: systolic blood pressure (SBP), diastolic blood pressure (DBP), mean arterial pressure (MAP).

We firstly analyzed the change in blood pressure after single oral administration with different doses of EPA-PL, as shown in Figure 1A. The high dosage of EPA-PL significantly reduced the SBP compared with the model group (*p* < 0.05), and the moderate dosage of EPA-PL appeared to decrease the SBP; however, there was no significant difference between the Moderate EPA-PL and M groups. The low dosage of EPA-PL failed to lower the level of SBP in SHRs (*p* > 0.05). Likewise, the high dosage of EPA-PL distinctly decreased the DBP compared with the model group (*p* < 0.05), and the moderate and low dosage of EPA-PL were not able to reduce the level of DBP (*p* > 0.05). MAP is defined as the average arterial blood pressure during a single cardiac cycle, which reflects the hemodynamic perfusion pressure of the vital organs. In the present study, the MAP in the High EPA-PL group was significantly reduced by 16 mmHg relative to the model group (*p* < 0.05). Dietary moderate and low dosage of EPA-PL had no effect on the decrease in MAP in SHRs (*p* > 0.05). The short-term plot experiment showed that the high dosage of EPA-PL had a positive antihypertensive effect in SHRs.

### 2.2. The Effects of EPA-PL on Growth Parameters in SHRs

In order to further illustrate the possible mechanism of EPA-PL on alleviating hypertension and hypertensive-induced renal interstitial fibrosis, SHRs were administrated with 2% EPA-PL for three weeks. The dosage of EPA in the diet was based on the recommendation of the human intake level, which was approximately equivalent to 3 g DHA + EPA/day [16]. After administration with EPA-PL for three weeks, there was no obvious difference in the food intake between the model group and EPA-PL group (*p* > 0.05, Figure 1B). In addition, we measured the change in blood pressure in the rats during the long-term experiment, as shown in Figure 1C–E. At the beginning of the study, there was a higher level of SBP, DBP, and MAP in the SHRs than the control group (*p* < 0.05). After one week of dietary intervention, EPA-PL failed to reduce the SBP in SHRs, interestingly, there was a lower level of SBP in the EPA-PL group after intervention for two weeks (*p* < 0.05), which was similar to the effect of the intervention for three weeks. Likewise, the level of DBP in EPA-PL group was remarkably reduced with the prolonging of time, especially after two weeks (*p* < 0.05). Moreover, dietary EPA-PL could distinctly reduce the MAP level after treatment with EPA-PL for two weeks. This indicates that the supplementation with EPA-PL has obvious effects on the prevention and treatment of hypertension. Moreover, the heart index and kidney index were significantly elevated in the model group compared to those in the control group, and there was a lower level of heart and kidney index in the EPA-PL group (*p* < 0.05, Figure 1F,G). 

### 2.3. The Effects of EPA-PL on The Renin–Angiotensin System in SHRs

**Figure 2:**
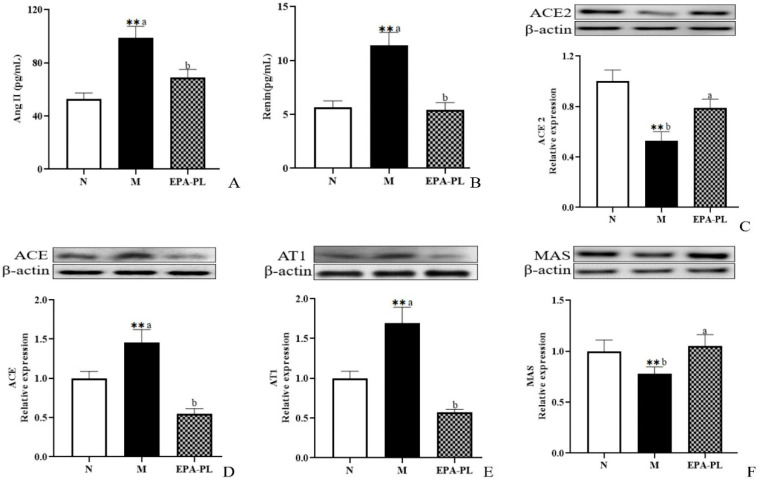
The effects of dietary EPA-PL on the concentration of the serum Ang II and renin in rats (**A**,**B**); expressions of ACE2, ACE, AT1, and MAS in the kidney were analyzed by Western blotting with β-actin used as the loading control of total proteins; the band density was quantified by scanning densitometry (**C**–**F**). All data are expressed as mean ± S.E.M. for 6 rats. ** Significantly different compared to control group (*p* < 0.01). Different letters represent significant differences between experimental groups, and means with the same letter are not significantly different (*p* < 0.05).

The renin–angiotensin system performs a vitally important role in maintaining the balance of blood pressure in the body. However, when the renin–angiotensin system (RAS) is pathologically activated, it could cause excessive vasoconstriction, proliferation and hypertrophy of vascular smooth muscle and myocardium, and even fibrosis [8]. Renin and angiotensin II are two important substances that reflect the degree of activation of the renin–angiotensin system. A high level of renin and Ang II in the serum was measured in the model group (Figure 2A,B). There was a lower content of renin and Ang II in the EPA-PL group compared with the model group. Yang et. al. found that treatments with marine-based *n*-3 fatty acids (2 g of EPA + DHA (EPA:DHA = 2:1)) had a notably lowering effect on Ang II in the plasma of Chinese hypertensive patients over a treatment period of 90 days [17].

Renin, as an angiotensinogenase, is secreted by the kidneys that participate in the renin–angiotensin system; therefore, the physiological function of the kidney is closely related to the regulation of blood pressure [18]. In the systemic effect on blood pressure regulation, ACE2 can degrade Ang II (vasoconstriction, pro-fibrosis, and pro-inflammation factor), and converts it into Ang (1–7) (vasodialatic and apoptotic factor). Moreover, ACE2 maintains the dynamic balance between the ACE/Ang II/AT1 pathway and the ACE2/Ang (1–7)/MAS receptor pathway in the RAS system [19]. Therefore, we next analyzed the change in RAS-related protein expression level in the kidney of rats (Figure 2C–F). Compared with the control group, the level of ACE2 was sharply reduced in the model group (*p* < 0.05), and the EPA-PL supplementation rapidly raised the expression level of ACE2 (*p* < 0.05). Both ACE and AT1 protein expression levels in the model group were significantly raised (*p* < 0.05), and the intervention of EPA-PL inhibited the protein expression levels of ACE and AT1 (*p* < 0.05). Moreover, dietary EPA-PL significantly raised the expression level of MAS compared with the model group (*p* < 0.05). Ulu et al. showed that the intake of DHA could increase the mRNA expression of ACE2 and AT1 levels in the kidney of mice with angiotensin II-dependent hypertension [20]. According to the above results, the nutritional intervention of EPA-PL had a strong protective effect against hypertension in SHRs through regulation of the RAS system.

### 2.4. The Effects of EPA-PL on Serum Parameters and Renal Pathological Changes in SHRs

**Figure 3:**
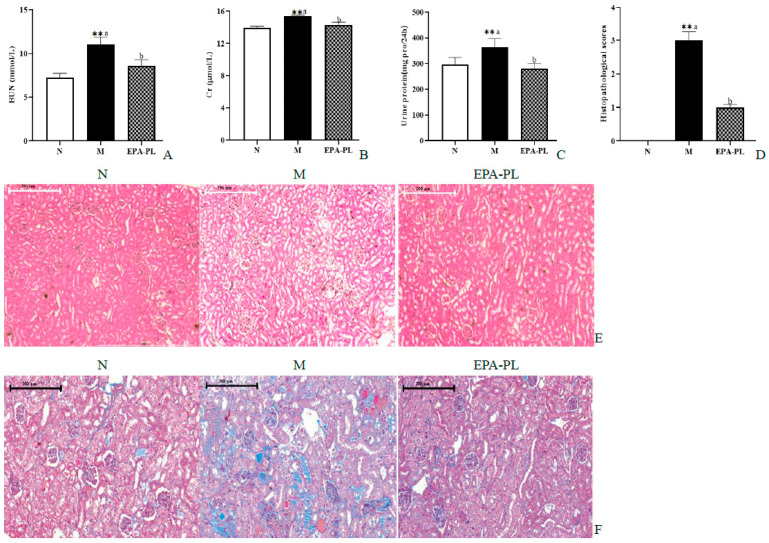
The effects of dietary EPA-PL on the level of the serum BUN, Cr, and 24 h proteinuria (**A**–**C**); results of total histopathological scores reflecting tubular damage in each group (**D**); representative pictures stained with H&E (**E**) and Masson trichrome (**F**) in different groups. All data are expressed as mean ± S.E.M. for 6 rats. ** Significantly different compared to control group (*p* < 0.01). Different letters represent significant differences between experimental groups, and means with the same letter are not significantly different (*p* < 0.05).

The BUN, Cr, and 24 h proteinuria are the main indexes of kidney function biomarkers in clinical diagnosis. We next measured the contents of BUN and Cr in the serum, as well as the 24 h proteinuria of the SHRs (Figure 3A–C). There was a significant increase in the levels of BUN, Cr, and the 24 h urinary protein compared to that of the control group (*p* < 0.05). These changes were consistent with previous research results [18]. This indicates that we successfully established a hypertensive nephropathy animal model in the present study. The administration of EPA-PL markedly decreased the levels of BUN, Cr, and the 24 h urinary protein (*p* < 0.05). Toroudi et al. demonstrated that the intervention of DHA + EPA for 14 days at a dose of 200 mg/kg body weight can clearly reduce the BUN and Cr contents in the serum of rats with kidney disease caused by ischemia–reperfusion [21]. Our previous study indicated that the administration of EPA-PL for seven days at a dose of 300 mg/kg body weight remarkably suppressed the elevated BUN and Cr levels in the mice with vancomycin-induced nephrotoxicity [22].

The H&E staining and Masson staining methods were used to analyze the pathological changes in the kidney for renal function assessment in clinical practices [23]. The pathological changes were observed in the kidneys of SHRs via an electron microscope, and the damage degree of the kidney was evaluated according to our previous methods (Figure 3D) [24]. Compared with the control group, there was significant glomerular atrophy, dilatation of tubules, or renal interstitial inflammation in the kidney tissue in the model group (Figure 3E). In fact, Masson staining showed that there was severe renal interstitial fibrosis in the kidney of the model group (Figure 3F). Expectedly, dietary supplementation with EPA-PL strongly ameliorated these pathological changes in the kidney, which was consistent with the results of the serum biochemical parameters in SHRs. Encarnacion et al. reported that treatment of fish oil for four weeks remarkably suppressed vascular hypertrophy, segmental and global glomerular sclerosis, interstitial fibrosis, and tubular atrophy in the kidney of rats with hypertension caused by excess dietary salt intake [25]. Additionally, some researchers have suggested that treatment of l-α-phosphatidylcholine for 30 days at a dose of 100 mg/kg body weight alleviated the kidney pathological injury of HgCl2-treated rats [26]. Likewise, our previous studies indicated that EPA-PL significantly inhibits the degree of kidney pathological injury in obesity-induced renal injury in mice. [14]. The above results indicate that EPA-PL has a prominent effect against hypertension-induced kidney damage.

### 2.5. The Effects of EPA-PL on TGF-β/Smad Signaling Pathway in SHRs

**Figure 4:**
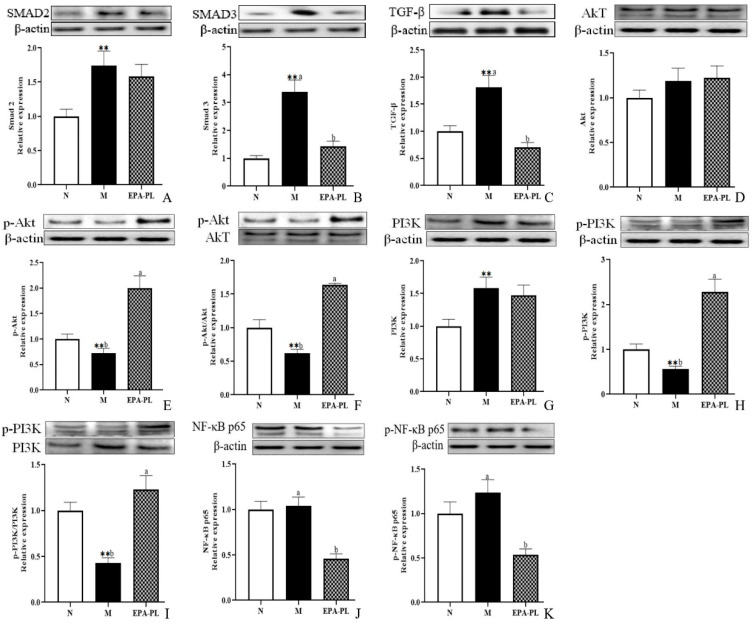
Expressions of Smad 2, Smad 3, TGF-β, Akt, p-Akt, p-Akt/Akt, PI3K, p-PI3K, p-PI3K/PI3K, NF-κB p65, and p-NF-κB p65 in the kidney were analyzed by Western blotting with β-actin used as the loading control of total proteins; the band density was quantified by scanning densitometry (**A**–**K**). All data are expressed as mean ± S.E.M. for 6 rats. ** Significantly different compared to control group (*p* < 0.01). Different letters represent significant differences between experimental groups, and means with the same letter are not significantly different (*p* < 0.05).

Emerging evidence indicates that renal interstitial fibrosis is a typical pathological feature in the development of hypertension-induced kidney damage [3]. In addition, renal interstitial fibrosis is the common pathway and the main pathological basis for almost all primary and secondary chronic kidney diseases to progress to end-stage renal disease [27]. Our current research has shown that there is strong renal interstitial fibrosis in the kidney of SHRs according to the results of Masson staining. It has been reported that the excessive activation of the TGF-β/Smad pathway has a significant role in inducing and aggravating renal interstitial fibrosis. Smad 2 and Smad 3 are the key molecules mediating TGF-β1 activity, leading to renal fibrosis [28,29]. As shown in Figure 4A,B, the protein expression of Smad 2 and Smad 3 in the kidney was higher in the model group than the control group (*p* < 0.05). Dietary EPA-PL clearly suppressed the expression of the Smad 3 protein (*p*< 0.05); however, there was no obvious difference in the protein expression of Smad 2 between the model and EPA-PL groups (*p* > 0.05). Moreover, there was a high expression of the TGF-β protein in the kidney in the model group (*p* < 0.05, Figure 4C). As expected, dietary EPA-PL clearly inhibited the expression of TGF-β (*p* < 0.05). Some reports have suggested that the pre-treatment of EPA significantly inhibited the expression of Smad 3 and TGF-β in renal epithelial cells (HK-2 cells) [29]. Cao et al. showed that dietary EPA-PL inhibited the expressions of TGF-β-activated kinase 1 in mice with chronic stress disease [30]. Additionally, some studies have verified that ursodeoxycholyl lysophosphatidylethanolamide (UDCA-LPE) could be suitable for the prevention of fibrotic progression of in the C57BL/6 mice with methionine–choline-deficient diet-induced chronic liver disease by blocking the TGF-β1/Smad 2/3 signaling pathway [31]. Our current study demonstrated that EPA-PL supplementation can inhibit the activation of TGF-β and Smad 3; however, the protein levels of Smad 2 revealed no changes following EPA-PL treatment. This inconsistent result might be related to the intervention time of the substance.

### 2.6. The Effects of EPA-PL on PI3K/Akt/NF-κB Signaling Pathway in SHRs

Accumulating evidence has demonstrated that the PI3K/Akt/NF-κB signaling pathway is closely related to fibrosis, which might be involved in the course of the disease, and previous studies have also found that TGF-β can regulate the phosphorylation of the PI3K protein [32]. Moreover, the PI3K/Akt signaling pathway was one of the upstream regulators for the NF-κB signaling pathway, and the NF-κB could promote the activation of Smad 3, and subsequently accelerate the fibrosis of tissues [33]. Compared with the control group, a distinctive decrease was found in the phosphorylation levels of PI3K and Akt of the model group (*p* < 0.05); furthermore, there was a higher level of p-PI3K and p-Akt expression after treatment with EPA-PL than in the model group (*p* < 0.05, Figure 4D–I). Interestingly, the expression level of PI3K was sharply elevated in the SHRs (*p* < 0.05); however, the expression level of Akt was not distinctively changed in the SHRs (*p* > 0.05). Moreover, the ratios of p-PI3K/PI3K and p-Akt/Akt in the EPA-PL group were drastically increased compared with the model group (*p* < 0.05). Furthermore, levels of both NF-κB p65 and p-NF-κB p65 were drastically reduced in the EPA-PL group compared with the model group (*p* < 0.05). Some researchers have reported that *n*-3 PUFA mediates a protective effect against LPS-induced PC12 cell injury by activating the PI3K/Akt pathway [34]. Additionally, EPA-PC treatment for eight weeks with 2500 mg/kg body weight contributed to the elevated expression of p-Akt in the liver of mice with high-fat diet-induced insulin resistance [35].

### 2.7. The Effects of EPA-PL on NF-κB-Meditated Inflammation Signaling Pathway in SHRs

**Figure 5:**
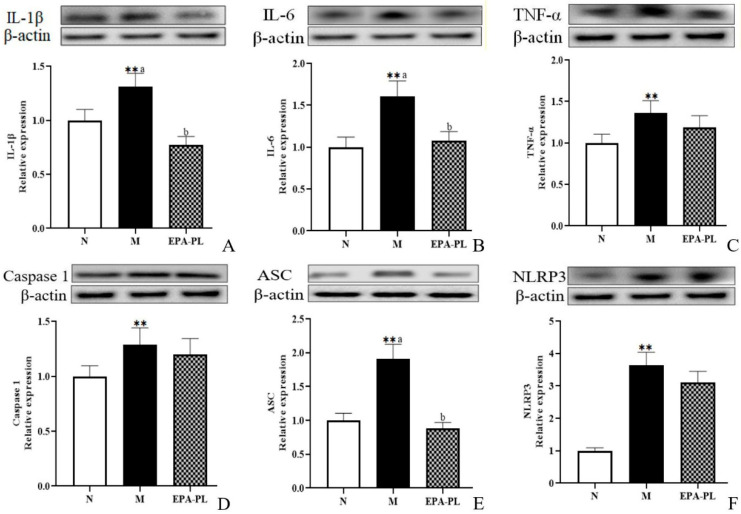
Expressions of IL-1β, IL-6, TNF-α, ASC, caspase-1, and NLRP3 in the kidney were analyzed by Western blotting with β-actin used as the loading control of total proteins; the band density was quantified by scanning densitometry (**A**–**F**). All data are expressed as mean ± S.E.M. for 6 rats. ** Significantly different compared to control group (*p* < 0.01). Different letters represent significant differences between experimental groups, and means with the same letter are not significantly different (*p* < 0.05).

Studies have reported that the activation of the inflammasome and subsequent release of proinflammatory cytokines are involved in renal interstitial fibrosis [36]. A previous study indicated that the inhibition of the NF-κB/IL-1β signaling pathway is involved in the prevention of unilateral ureteral obstruction-induced renal fibrosis in mice, especially, NF-κB can promote the activation of Smad 3 [37]. The histopathology analysis showed inflammatory infiltration in the model group, which might be related to a variety of inflammatory cells in the kidney, such as monocyte macrophages, T-lymphocytes, or neutrophils. Therefore, we focused on the changes in inflammation-related protein levels in the whole kidney [38]. As shown in Figure 5, both IL-1β and IL-6 protein levels were sharply increased in the SHRs compared to those of the control group (*p* < 0.05), and dietary EPA-PL clearly reduced the expression levels of IL-1β and IL-6 proteins (*p* < 0.05). TNF-α is a strong pro-inflammatory cytokine that plays an important role in the immune system during inflammation. An increasing number of studies have shown that the NLRP3–ASC–caspase-1 inflammasome is a novel innate immune sensor and pro-inflammatory cytokine, and the NLRP3 inflammasome-mediated caspase-1 activation is a key factor in the development of kidney diseases. In the current study, there was a higher level of TNF-α in the model group compared with the control group (*p* < 0.05), and the intervention of EPA-PL failed to inhibit the expression of TNF-α (*p*> 0.05). Similarly, the supplementation of EPA-PL was not able to reduce the abnormal rise in caspase-1 protein level (*p* > 0.05). Moreover, the expression of ASC and NLRP3 was significantly increased in the model group (*p* < 0.05), and the intervention of EPA-PL distinctly inhibited the expression of ASC (*p* < 0.05); however, there was no significant difference for NLRP3 between the model group and EPA-PL group (*p* > 0.05). Emerging evidence indicates that EPA possesses high anti-inflammatory activity. Dietary supplementation of EPA at the dose of 400 mg/kg body weight by gavage for 63 days significantly suppressed the increase in IL-1β and TNF-α expression levels in the intestinal epithelium of dextran sulphate sodium (DSS)-induced colitis mice [39]. Moreover, the concentration of pro-inflammatory cytokines, such as TNF-α, significantly decreased in the serum of the SHRs after the intervention of the lecithin derived from ω-3 PUFA fortified eggs [40]. Our previous research found that EPA-PL alleviated atherosclerosis by inhibiting the transcriptional levels of IL-6 and TNF-α in ApoE^−/−^ mice [41]. In addition, dietary intervention with EPA-enriched phosphoethanolamine plasmalogen can attenuate neurodegenerative diseases by reducing the levels of IL-1β and caspase-1 in the brain of rats with Alzheimer’s disease. [42]. Some studies have shown that NF-κB not only activates the NLRP3 inflammasomes, it also induces the activation of the mitogen-activated protein kinase (MAPK) signaling pathway, and then regulated downstream protein IL-1β precursor and IL-1β signal transduction [33]. Our present results indicate that the expression of caspase-1 and NLPR3 is not substantially reduced in the EPA-PL group. We speculate that EPA-PL supplementation might inhibit the pro-inflammatory cytokines (IL-1β and IL-6) by regulating the MAPK signaling pathway. This will be one of the directions that we will continue to focus on in future experiments.

### 2.8. The Effects of EPA-PL on Oxidative Stress in SHRs

**Figure 6:**
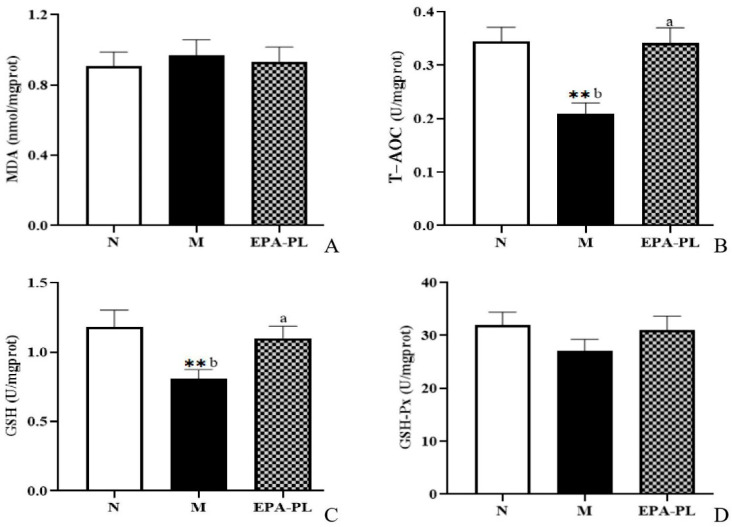
The levels of MDA, T-AOC, GSH, and GSH-Px in the kidneys of the three groups of rats (**A**–**D**). All data are expressed as mean ± S.E.M. for 6 rats. ** Significantly different compared to control group (*p* < 0.01). Different letters represent significant differences between experimental groups, and means with the same letter are not significantly different (*p* < 0.05).

Recent evidence suggests that oxidative stress is one of the major contributing factors in the development of hypertension. Indeed, some studies have shown that there is a strong link among oxidative stress, NF-κB-meditated inflammation, and interstitial fibrosis in the kidney [4]. MDA, T-AOC, GSH, and GSH-Px are common indicators of oxidative stress [28]. In the current experiment, as shown in Figure 6, both MDA and GSH-Px activity showed no significant difference among these groups (*p* > 0.05). The enzyme activity of T-AOC was significantly reduced in the kidney of the model group compared with the control group, and the EPA-PL supplementation remarkably increased the T-AOC activity (*p* < 0.05). Similarly, the enzyme activity of GSH was clearly decreased in the kidney of the model group to a greater degree than in the control group (*p* < 0.05), and dietary EPA-PL sharply elevated the GSH activity in the kidney (*p* < 0.05). Our findings clarify that EPA-PL can inhibit the oxidative stress in the kidney, and thus protect against renal interstitial fibrosis. In accordance with our findings, some studies have shown that the administration of EPA (300 mg/kg per day for two weeks) clearly suppressed MDA activity and elevated the activity of GSH, in the liver of rats with valproate-induced liver toxicity [43]. Likewise, our previous results showed that the intake of EPA-PL for 10 days markedly reduced the level of MDA, and elevated the activity of antioxidant enzymes in the kidneys of mice with vancomycin-induced nephrotoxicity [22]; and the intervention of EPA-PL significantly decreased the level of MDA and elevated the GSH and GSH-Px activity in the brains of mice with Alzheimer’s disease [42].

### 2.9. The Effects of EPA-PL on Mitochondria-Mediated Apoptosis Signaling Pathway in SHRs

**Figure 7:**
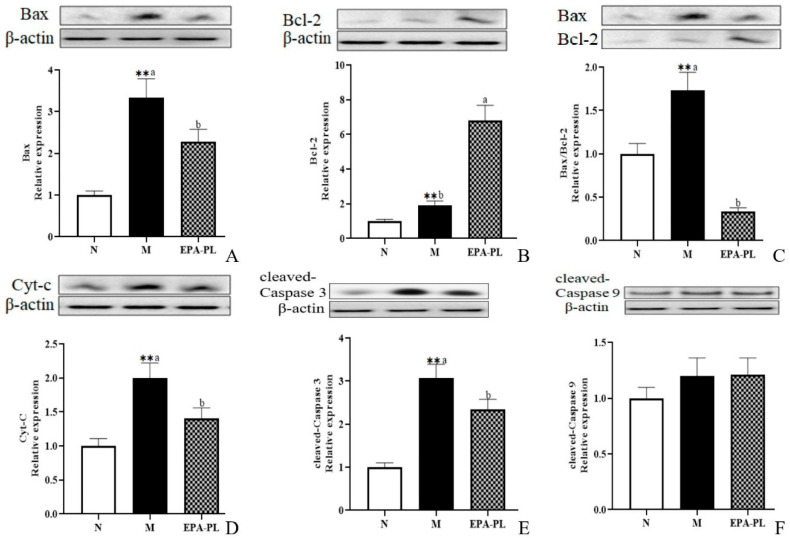
Expressions of Bax, Bcl-2, Bax/Bcl-2, Cyt-C, cleaved caspase-3, and cleaved caspase-9 in the kidney were analyzed by Western blotting with β-actin used as the loading control of total proteins; the band density was quantified by scanning densitometry (**A**–**F**). All data are expressed as mean ± S.E.M. for 6 rats. ** Significantly different compared to control group (*p* < 0.01). Different letters represent significant differences between experimental groups, and means with the same letter are not significantly different (*p* < 0.05).

A plethora of studies have suggested that apoptosis plays an important role in the development of kidney injury, and that the activation of the apoptosis signaling pathway can further promote renal interstitial fibrosis [44,45]. There is conclusive evidence that Bax and Bcl-2 are the major mediators of endogenous apoptosis, and that apoptosis is activated by Bax and inhibited by Bcl-2 [27]. The occurrence of apoptosis might be attributed to multiple types of cells in the kidney, such as the endothelial cells, podocytes, mesangial cells, immune cells, and renal tubular epithelial cells [46,47]. Therefore, we focused on the changes in proteins associated with apoptotic signaling pathway in the whole kidney. First, we examined the expression of pro-apoptotic and anti-apoptotic proteins in the kidney (Figure 7A–C). The expression levels of pro-apoptotic protein Bax were significantly increased in the SHRs compared with the control group (*p* < 0.05), and the increase was clearly reduced after the EPA-PL supplementation (*p* < 0.05). Interestingly, the expression levels of anti-apoptotic protein Bcl-2 were sharply raised in the model group more than in the control group, which might be related to the role of organism self-protection after kidney injury; in addition, the level of Bcl-2 in the EPA-PL group was higher than the model group (*p* < 0.05). In the current study, the ratio of Bax/Bcl-2 was significantly increased in the model group relative to the normal group, and EPA-PL administration sharply reduced the ratio of Bax/Bcl-2 when compared with the model group (*p* < 0.05). The increase in the Bax/Bcl-2 ratio could stimulate the release of cytochrome c (Cyt-C) from the mitochondria to the cytoplasm, and then activate caspase-9. At the same time, it has been reported that the inhibitor of apoptosis proteins (IAPs) are a family of proteins that function as intrinsic regulators of the caspase cascade, and may have a crucial role in the inflammatory response in kidney disease [48]. Sebe et al. found that a diet enhanced with EPA and DHA resulted in cooperative protection against 5-fluorouracil-induced mucosal impairment by up-regulating the expression of BCL-2 and IAP-1 genes in mice [49]. In the present study, we speculated that EPA-PL supplementation might increase the expression of IAP-1, which needs to be further studied. Oxidative stress may induce the activation of NF-κB/NLRP3/caspase-1 pathway pyroptosis and intrinsic apoptosis, which induces the release of Cyt-C before activating caspase-9 and downstream caspase-3, eventually triggering apoptosis [28,50]. Notably, there was a higher level of Cyt-C protein in the model group (*p* < 0.05, Figure 7D); as expected, the intervention of EPA-PL remarkably suppressed the protein expression of Cyt-C in the kidney (*p* < 0.05). The expression levels of cleaved caspase-3 protein were increased in the SHRs more than in the control group, and EPA-PL significantly inhibited the expression of cleaved caspase-3 in the kidneys of the rats (*p* < 0.05). Furthermore, the expression of cleaved caspase-9 protein showed a rising tendency in the model group, although the results show no significant difference among all groups (*p* > 0.05). Emerging evidence indicates that the intervention of EPA (a standard chow supplemented with 5% EPA) for two weeks can clearly inhibit the expression of Cyt-C, cleaved caspase-3, and cleaved caspase-9 proteins in the kidney of mice with diabetic tubular injury [50]. Similarly, our previous studies have found that the administration of EPA-PL (300 mg/kg body weight) for seven days markedly decreased the protein expression of Bax and cleaved caspase-3 in the kidneys of mice with acute kidney injury [22], as well as suppressed the protein expression of Bax, caspase-3, and cleaved caspase-3, and elevated the level of Bcl-2 protein, in the brains of rats with Alzheimer’s disease [42].

The Mediterranean diet comprises a high intake of fruits, vegetables, olive oil, unrefined grains, legumes, and fish; a moderate intake of nuts and red wine; and a low intake of red meat and refined sugar [51]. Consumption of marine *n*-3 PUFAs from fish and other seafood is high in certain Mediterranean countries. Dietary Approaches to Stop Hypertension (DASH) is a dietary pattern rich in fruits, fish, vegetables, and low-fat dairy products, and is low in meats and sweets [52]. Nutritional epidemiology studies and clinical trials confirm the salutary effects of Mediterranean diet and DASH on the prevention of coronary artery disease, stroke, and dementia [52]. Epidemiological studies and controlled trials indicate that plant- and sea-derived *n*-3 PUFAs are likely to be important mediators of the protection provided by traditional Mediterranean diets and DASH due to their anti-inflammatory properties [51]. Our results suggest that *n*-3 PUFAs in the Mediterranean diet or DASH might be active ingredients for the prevention of hypertensive nephropathy.

At present, there are still inconsistent views on the hypotensive effect of EPA in clinical studies. On the one hand, numerous studies have reported that EPA treatment has a strong antihypertensive effect; on the other hand, some research indicates that EPA intervention has no significant effect on blood pressure [9,53]. Moreover, new research shows that EPA treatment minimizes inflammation and oxidative stress in rats with type 2 diabetes mellitus, the effects of which were associated with less glomerular sclerosis and less interstitial fibrosis; however, EPA supplementation was unable to decrease the abnormally elevated blood systolic pressure in diabetic rats [54]. We speculate that these effects might be related to the time of intervention and EPA dose, as well as the form of EPA. However, although the present study suggests that the supplement of EPA-PL has a significant protective effect on hypertensive nephropathy, there are still some problems that have not been completely illustrated. It is necessary to further explore the effects of phospholipids containing different polar heads on hypertensive nephropathy in future research. Additionally, the efficacy differences between other active substances and EPA-PL have not yet been compared, especially for traditional EPA and positive drugs. This should be the focus of future research.

## 3. Materials and Methods

### 3.1. Materials

Sea cucumber (*Cucumaria frondosa*) was purchased from the Nanshan aquatic products market (Qingdao, Shandong, China). The enzymatic reagent kit of creatinine (Cr), blood urea nitrogen (BUN), malondialdehyde (MDA), total antioxidant capacity (T-AOC), glutathione (GSH), and glutathione peroxidase (GSH-Px) were obtained from Nanjing Jiancheng Bioengineering Institute (Nanjing, Jiangsu, China). The enzyme-linked immunosorbent assay kits for Angiotensin II (Ang II) and renin were provided by Shanghai Elisa Biotech Co., Ltd. (Shanghai, China). All primary antibodies, β-actin, and secondary antibodies were obtained from Abcam (Cambridge, MA, USA) and Cell Signaling Technology (Danvers, MA, USA).

### 3.2. Preparation of EPA-PL

The crude lipid was extracted from the body wall of the sea cucumber according to the modified method of *Folch*, and then the neutral lipid and glycolipid of the crude lipid were removed by cold acetone [55]. Additionally, the purity and the fatty acid composition of EPA-PL was determined by high-performance liquid chromatography coupled with evaporative light scattering detection (HPLC-ELSD) and gas chromatography (GC) according to the previous method [24]. The purity of EPA-PL was 95.6%, and the fatty acid compositions are shown in Table 1.

### 3.3. Animal Experiment Design

Male spontaneously hypertensive rats (SHR) (11 weeks old) and Sprague Dawley rats (SD, male, 11 weeks old) were fed under the conditions of 23 °C ± 2 °C, 12 h light/dark cycle, relative humidity 65% ± 5%. All procedures were approved by the Ethical Committee of the College of Food Science and Engineering, Ocean University of China (Qingdao, China, approval no. SPXY20200518, approved on 18 May 2020). Each rat was fed in a separate cage.

SHRs were randomly divided into four groups (*n* = 6 each group) after one week of adaptive feeding. They were designated as: model group (M), low dose of EPA-PL treated group (Low EPA-PL, orally gavaged with 250 mg/kg body weight (BW) EPA-PL), medium dose of EPA-PL treated group (Moderate EPA-PL, orally gavaged with 500 mg/kg BW EPA-PL), and high dose of EPA-PL treated group (High EPA-PL, orally gavaged with 750 mg/kg BW EPA-PL). The SBP, DBP, and MAP of the rats were evaluated as previously performed after the single administration of EPA-PL [35].

Moreover, the effects of EPA-PL with long-term intervention in SHRs were explored. The SHRs were randomly divided into two groups (six rats in each group): the model group fed with a high-fat diet (M, 45 kcal% from fat) and another group fed with a high-fat diet plus 2% EPA-PL (EPA-PL). Additionally, the Sprague Dawley (SD) rats were used as the control group (N). The ingredients in diets are shown in Table 2. The blood pressure of the rats was measured once a week at a fixed time. All rats were fasted for 12 h and euthanized after three weeks of continuous treatment. Blood, kidney, and heart tissues were collected from the rats, and stored in −80 °C refrigerators for future research.

### 3.4. Biochemical Analysis

Serum was collected from the blood by centrifugation at 4500× *g* for 15 min and then stored at −80 °C. The levels of serum renin, Ang II, Cr, and BUN were measured according to the instructions of the corresponding commercial kits. The kidney was homogenized with cold saline and centrifuged at 3000× *g* for 10 min to obtain supernatant for future determination. The activities of MDA, T-AOC, GSH, and GSH-Px were determined according to the instructions of the commercial kit.

### 3.5. Histological Analysis

The kidney tissue was fixed and dehydrated with 4% paraformaldehyde, then the tissue was embedded in paraffin and eventually stained with hematoxylin and eosin (HE) or Masson trichrome. Histological changes in the kidneys were evaluated and graded according to the method outlined in the previous report [24]. Renal pathological injury includes tubular necrosis, renal fibrosis, inflammatory infiltration, and tubular atrophy or expansion. Scoring criteria were: 0 (no renal tubular lesion), 1 (mild injury, renal tubular injury < 10%), 2 (moderate injury, renal tubular injury < 25%), 3 (severe injury, renal tubular injury 26−50%), 4 (very severe injury, renal tubular injury > 51%).

### 3.6. Western Blotting Analysis

Kidney tissue was homogenized by radio-immunoprecipitation assay (RIPA) buffer according to the methods of published research [24]. The protein concentration was determined using a bicinchoninic acid assay (BCA) commercial kit. The proteins of Smad 2, Smad 3, transforming growth factor-β (TGF-β), angiotensin-converting enzyme (ACE), angiotensin-converting enzyme 2 (ACE2), MAS, angiotensin type 1 receptor (AT1), Bax, Cytochrome C (Cyt-C), Bcl-2, caspase-3, cleaved caspase-3, cleaved caspase-9, IL-1β, IL-6, NF-κB p65, p-NF- κB p65, Akt, p-Akt, PI3K, and p-PI3K were analyzed as previously performed [24]. The protein expression was evaluated using anti-β-actin antibody (1:1000).

### 3.7. Statistical Analysis

All data are presented as mean ± standard error of the mean (S.E.M). A value of *p* < 0.05 was considered statistically significant. The Student’s t-test was used to determine the difference between the control group and model group, and the differences among the SHRs groups (M, EPA-PL) were determined and analyzed by one-way analysis of variance (ANOVA) and Tukey’s post hoc test. Different letters represent significant differences among experimental groups, and means with the same letter are not significantly different (*p* > 0.05).

## 4. Conclusions

In summary, the present results indicate that the supplementation of EPA-enriched phospholipids could effectively relieve hypertension by regulating the renin–angiotensin system in spontaneously hypertensive rats. Moreover, dietary EPA-PL could alleviate hypertensive nephropathy by inhibiting renal interstitial fibrosis. Further mechanistic studies showed that EPA-PL ameliorated renal interstitial fibrosis by inhibiting TGF-β/Smad-meditated tubulointerstitial fibrosis, activating the PI3K/Akt/NF-κB signaling pathway, regulating oxidative stress, and inhibiting the mitochondria-mediated apoptosis pathway. These findings indicate that EPA-PL may be used as a functional food ingredient for the prevention of hypertensive nephropathy.

## Figures and Tables

**Table 1:** Main fatty acid compositions of EPA-PL.

Fatty Acids	EPA-PL (g/100 g)
C14:0	3.41 ± 0.23
C16:0	3.63 ± 0.29
C16:1 *n*-9	6.69 ± 0.75
C18:0	5.32 ± 0.48
C18:1 *n*-9	5.45 ± 0.36
C20:1 *n*-9	6.93 ± 0.59
C20:3 *n*-6	1.95 ± 0.12
C20:3 *n*-3	5.75 ± 0.41
C20:5 *n*-3 (EPA)	38.61 ± 2.91
C22:6 *n*-3 (DHA)	4.21 ± 0.38

**Table 2:** Ingredients of the experimental diets (N, M, EPA-PL).

Ingredients (g/kg)	N	M	EPA-PL
Potato starch	349.5	349.5	349.5
Casein	200	200	200
Sucrose	100	100	100
Soy oil	50	50	46
Lard	200	200	184
Cellulose	50	50	50
Mineral–salt mix	35	35	35
Vitamin mix	10	10	10
l-cystine	3	3	3
Choline bitartrate	2.5	2.5	2.5
EPA-PL	-	-	20

Note: -, none added.

## Data Availability

Not applicable.
